# Gestational and congenital syphilis in the state of Rio de Janeiro, Brazil, 2021‒2023

**DOI:** 10.1016/j.bjid.2025.104522

**Published:** 2025-03-28

**Authors:** Rosa Maria Soares Madeira Domingues, Marcos Augusto Bastos Dias, Ana Paula Esteves Pereira, Paula Mendes Luz, Emilia M. Jalil, Angela Cristina Vasconcelos de Andrade Rabello, Ruth Khalili Friedman, Maria do Carmo Leal

**Affiliations:** a Fundação Oswaldo Cruz, Instituto Nacional de Infectologia Evandro Chagas, Laboratório de Pesquisa Clínica em HIV, Aids, Infecções Sexualmente Transmissíveis e Hepatites, Rio de Janeiro, RJ, Brazil; b Fundação Oswaldo Cruz, Instituto Nacional de Saúde da Mulher, da Criança e do Adolescente Fernandes Figueira, Rio de Janeiro, RJ, Brazil; c Fundação Oswaldo Cruz, Escola Nacional de Saúde Pública Sérgio Arouca, Rio de Janeiro, RJ, Brazil

**Keywords:** Syphilis, Pregnancy, Congenital syphilis, Prenatal care, Vertical transmission of infectious disease, Observational study, Brazil

## Abstract

Gestational (GS) and congenital syphilis (CS) are important public health problems in Brazil. This study aims to estimate the prevalence of GS, the incidence of CS and the rate of vertical transmission (VT) of syphilis, as well as to evaluate the management indicators of GS in the State of Rio de Janeiro (RJS), the Brazilian state with the highest detection rate of GS and incidence of CS in 2022. A hospital-based, cross-sectional study was carried out in public and private hospitals located in RJS, in the period 2021–2023, with interviews with 1,923 women, analysis of prenatal care (PNC) cards and hospital records. The GS management indicators, the prevalence of GS, the incidence of CS and the rate of VT were estimated with the respective 95 % confidence intervals (95 % CI), according to the source of financing for hospitalizations for childbirth or abortion care. PNC was reported by 93.7 % of women, 82.7 % had the first test for syphilis and 52.6 % the second. The prevalence of GS was estimated at 14.5 % (95 % CI 9.2 %- 22.2 %), with higher values in women with public financing (18.2 % public; 3.6 % private). Nearly one-third-of women with GS were diagnosed only during hospitalization for childbirth or abortion care and 13.4 % were appropriately treated during PNC. The incidence of CS was estimated at 53.1 per 1,000 live births (68.4 per 1,000 public; 9.7 per 1,000 LB private) with a VT rate of 33.5 %, with no difference according to the source of financing. The detection rate of GS and the incidence rate of CS were double those reported to the Brazilian Notifiable Diseases Information System. Several missed opportunities for the control of CS were identified. Women with public financing had a higher prevalence of GS and incidence of CS, and should be the priority target of control strategies.

## Introduction

Congenital Syphilis (CS) is an important global health problem, which can be avoided through adequate management of Gestational Syphilis (GS).^1^^,^^2^ It is estimated that almost one million cases of GS occurred worldwide in 2016, with several associated negative outcomes, including prematurity, low birth weight, miscarriages, stillbirths and neonatal deaths.^1^

In 2014, the World Health Organization (WHO) defined a global goal of eliminating CS, translated by the occurrence of ≤ 0.5 cases of CS per 1000 Live Births (LB). To achieve this goal, the WHO recommends at least one Prenatal Care (PNC) consultation for ≥95 % of pregnant women; serological testing to diagnose syphilis infection during pregnancy for ≥95 % of pregnant women undergoing PNC; and adequate treatment of ≥95 % of infected pregnant women.^3^

In Brazil, several CS elimination strategies have been adopted since the 1990s, the most recent being the certification of municipalities with >100,000 inhabitants that achieve elimination targets.^4^ Despite these efforts, both GS and CS have showed an increase in cases in the country. The GS detection rate has been growing, with an acceleration between 2020 and 2022, reaching a value of 32.5 per 1000 LB. Although the incidence of CS remained stable between 2021 and 2022, with a value close to 10 per 1000 LB, there was an increase of 16 % between 2019 and 2022.^5^

The State of Rio de Janeiro (RJS), the third most populous state in Brazil, located in the Southeast region of the country, presented the highest detection rate of GS and the highest incidence rate of CS in the country in 2022, with values of 69.7 per 1000 LB and 23.0 per 1000 LB for GS and CS, respectively.^5^ Previous studies carried out in the city of Rio de Janeiro, capital of the state, demonstrate missed opportunities in preventing vertical transmission of syphilis, as well as failures in case notification.^6^, ^7^, ^8^ Previous studies also demonstrate social inequalities in the prevalence of GS, access to health services and the incidence of CS,^9^, ^10^, ^11^, ^12^, ^13^ with worse indicators in women treated in public services.^9^

This study aims to estimate the prevalence of GS, the incidence of CS and the rate of vertical transmission of syphilis in RJS, as well as to evaluate the management indicators of GS, according to the source of financing for hospitalization for childbirth or abortion care, aiming to provide support for the development of strategies for the control of GS and CS.

## Material and methods

This is a national, hospital-based, cross-sectional study, carried out in the period 2021‒2023 (Birth in Brazil II: National Research on Abortion, Labor and Childbirth).

### The birth in Brazil II research (NBII)

In the NBII, a two-stage probabilistic sample was selected: 1-Hospitals and 2-Women and their newborns. Hospitals were stratified by macro-region, location (metropolitan or interior region), type (public, mixed, private) and size (100‒499, ≥500 LB/year). In each hospital, 30 postpartum women were interviewed in hospitals with 100‒499 LB/year and 50 in hospitals with ≥500 LB/year. The sample size of women hospitalized for abortion was not fixed and corresponded to the number of hospitalizations for abortion that occurred in each hospital until reaching the planned sample of postpartum women.

Postpartum women with LB with any weight or Gestational Age (GA), postpartum women with stillbirths with GA ≥ 22 weeks or weight ≥ 500*g* , and women hospitalized with a diagnosis of early fetal loss were eligible. Women who gave birth outside the hospital institution, and/or had a triplet or more, and/or had difficulty communicating (foreigners, indigenous people who did not understand Portuguese, deaf/mute, or had severe mental illness), and/or those hospitalized with a diagnosis of abortion but who were discharged while still pregnant, were excluded.

For all women included in the study, interviews were carried out in the immediate postpartum/post abortion period and data was extracted from hospital records and PNC cards, when available. The NBII study protocol is published in Leal et al.^14^

### The study in the state of Rio de Janeiro

During the NBII study, the sample size of postpartum women in the state of Rio de Janeiro was calculated based on the proportion of cesarean sections in RJS in 2019 (57 %), a significance level of 5 % and power of 90 % to detect differences of 7 %. A design effect of 1.3 was used, resulting in the planning of a minimum sample size of 1350 postpartum women. To achieve this quantity, the sample was expanded from 50 to 90 postpartum women in public and mixed hospitals with ≥ 500 LB/year. The hospital interviews took place between November 2021 and June 2023 in 29 hospitals across 18 municipalities.

### Variables and data source

To evaluate the management of syphilis during pregnancy, the following process and outcome indicators were analyzed based on recommendations from the WHO^3^^,^^15^ and the Brazilian Ministry of Health^16^^,^^17^:

a) PNC: having received at least one PNC consultation;
b) Receipt of the PNC card (card containing records of exams, medications and procedures performed during the PNC);
c) Early start of PNC: having had the first PNC consultation up to the 12th gestational week;
d) Adequacy of the number of PNC consultations: appropriate number of consultations for GA at childbirth according to the following calendar^15^: GA ≥ 20 and < 26 = consultations ≥ 2; GA ≥ 26 and < 30 = consultations ≥ 3; GA ≥ 30 and < 34 = consultations ≥ 4; GA ≥ 34 and < 36 = consultations ≥ 5; GA ≥ 36 and < 38 = consultations ≥ 6; GA ≥ 38 and < 40 = consultations ≥ 7; GA ≥ 40 = consultations ≥ 8;
e) Testing for syphilis during pregnancy: (1) First test carried out at the beginning of PNC and (2) Second test from the 28th week of pregnancy. The adequacy of the first and second tests was assessed separately. For the second test, only postpartum women with GA ≥34 weeks at the time of delivery were considered. The absence of test records on the PNC card was classified as lack of testing during PNC;
f) Testing for syphilis during hospitalization for childbirth or abortion care: testing for syphilis during hospitalization. The absence of test records on the hospital records was classified as lack of testing during hospitalization;
g) Type of test used: Non-Treponemal Test (NTT), Treponemal Test (TT), or both. The NTT and TT mostly used in routine prenatal and hospital care were VDRL (Venereal Disease Research Laboratory) and rapid tests, respectively;
h) Prevalence of syphilis during pregnancy: cases of syphilis during pregnancy were classified according to the type of diagnosis (laboratory and/or clinical) and the moment of diagnosis (during PNC or hospitalization). To define the laboratory diagnosis, an algorithm was developed considering the combination of results from serological tests carried out in the three testing moments (first and second testing during PNC and testing during hospitalization). For the combined interpretation of the three testing moments, we considered the criteria described in Table 1, with cases classified as “confirmed laboratory diagnosis”, “probable laboratory diagnosis”, “diagnosis dependent on clinical history”, “probable false positive”, and “negative”. The “Diagnosis dependent on clinical history” category refers to women with NTT with low titers (< 1/8) + reactive TT, or only reactive TT with NTT not available, situations in which the history of previous treatment is relevant to the case definition. Cases classified as “exclusively clinical criterion” were those in which there was a record of syphilis diagnosis during PNC care, and/or during hospitalization for childbirth or abortion care, and/or the record of CS cases in the absence of information on laboratory diagnosis;

**Table 1:** Operational definition of laboratory diagnosis of Gestational Syphilis.

Test results for syphilis considering two prenatal tests and testing during hospitalization	Interpretation
Non-treponemal test with titer ≥1:8 and reactive treponemal test considering information available in the three testing moments	Confirmed diagnosis
Incident case: reactive treponemal test or non-treponemal test ≥1:8 in the second prenatal test after a first non-reactive test	Confirmed diagnosis
Incident case: reactive treponemal test or non-treponemal test ≥1:8 during hospitalization following results of non-reactive prenatal test	Confirmed diagnosis
Non-treponemal test ≥1:8 and non-reactive treponemal test or no result, except in the situations described above (incident cases)	Probable diagnosis
Reactive treponemal test at any of the testing moments, except in the situations above.	Dependent on clinical history
Non-treponemal test < 1:8 or with unknown titer and treponemal test not performed	Probable false-positive
Combination of non-reactive tests or tests not performed in any of the three moments or non-treponemal test with titer < 1:8 or titer not reported and non-reactive treponemal test	Without infection
Test not performed in any of the three moments	No laboratory diagnosis
i) Incidence of GS: cases of women with seroconversion during PNC follow-up or who presented a clinical or laboratory diagnosis during hospitalization, without prior record of infection during pregnancy. Cases with NTT tests with titer < 1:8 (or reactive but without titer value) and TT not performed were not considered for the classification of incident cases, due to the probability of being false-positive cases (Table 1);
j) Treatment for GS: women were classified as “not treated”, “adequate treatment”, “inadequate treatment”, “no information on adequacy”, “diagnosis during hospitalization” or “no information”. Women diagnosed during pregnancy and without a record of treatment on the PNC card were classified as “not treated”. Cases with treatment carried out with penicillin, started up to 30-days before the pregnancy termination, with 2400,000 U for cases of primary, secondary or recent latent syphilis (less than one year of infection) and with 7200,000 U (three doses of 2400.000 U at a weekly interval) for cases of late latent, tertiary or syphilis of unknown duration of infection were classified as “adequate treatment”. Cases with non-penicillin treatment, and/or an inadequate dose for the stage of the disease, and/or instituted <30 days before the pregnancy termination were classified as “Inadequate treatment”. Pregnant women treated, but with no record of information about the type of treatment and/or moment of institution, were classified as “no information on adequacy”. Pregnant women diagnosed during pregnancy and without PNC card were classified as “no information”;
k) Partner treatment: record of treatment of the pregnant woman's partner diagnosed with syphilis (no, yes, diagnosis during hospitalization, no information);
l) Incidence of CS: number of CS cases divided by the number of LB multiplied by 1000. Two incidence rates were calculated:
  l.1) incidence of recorded CS cases = cases recorded in hospital medical records (newborns diagnosed with CS or cases of neonatal or fetal death with CS recorded as the cause);
  l.2) estimated incidence of CS = CS cases recorded in hospital records plus probable CS cases, based on the CS case definition adopted by the Brazilian Ministry of Health (every outcome of the pregnancy of a woman diagnosed with GS with no treatment or inadequate treatment)^16^^,^^17^;
m) Type of CS case outcome: “live birth” (until hospital discharge), “early fetal loss” (fetal death weighing < 500 grams and GA < 22 weeks); “intermediate or late fetal loss” (fetal death weighing ≥ 500 grams or GA ≥ 22 weeks) and “neonatal death” (death occurring up to the 27th day of life). The outcomes of both registered and estimated CS cases were analyzed.

The following variables were analyzed as maternal characteristics: “type of hospital financing” (public = hospitalizations in public or private hospitals paid for with public funding; and private = hospitalizations in private hospitals paid by health plans or out-of-pocket payment); age (< 20-years, 20 to 34, 35 or more); race/skin color (white, brown and black), years of education (≤ 8, 9 to 11, 12 to 15 and 16 or more) and marital status (currently living with a partner or not).

All data relating to maternal characteristics and PNC card receipt, time of initiation of PNC and number of consultations were obtained during hospital interview with the women, while data relating to tests, treatment and pregnancy outcomes were obtained from the PNC card and hospital records.

### Data analysis

All analysis were categorized according to the type of hospital financing (public or private). Initially, we analyzed the demographic and social characteristics of the women. Then, we estimated the indicators for the management of syphilis during pregnancy with the respective 95 % Confidence Intervals (95 % CI). We used the reference standard ≥ 95 %, recommended by the WHO for process indicators to prevent vertical transmission of syphilis and HIV.^3^ For the result indicator “Incidence of congenital syphilis”, we used as reference the global elimination target established by the WHO in 2014, defined as the occurrence of ≤ 0.5 cases of CS per 1000 live births.^3^

All analysis were performed with the IBM SPSS Statistics for Windows, Version 19.0 software (IBM Corp., Armonk, NY, USA). We used weighting, calibration and design effect due to the complex sampling process. For the RJS sample, we used groups composed of the combination of stratum, type of birth (vaginal, cesarean section) and woman's age (10‒19, 20‒34, ≥ 35) for calibration, using data from the Live Birth Information System (SINASC), year 2022, as a reference.

The Birth in Brazil II study was approved by the National Research Ethics Commission (CONEP), CAAE: 21,633,519.5.0000.5240, on March 11, 2020, and was approved by the local research ethics committees of the institutions or the clinical board when local committees were absent. All women signed the free and informed consent form before the interview.

## Results

We interviewed 1923 women (1762 postpartum, 161 post abortion), 24.8 % of whom had private financing. Most of the women were aged 20 to 34, were brown, had 12 or more years of education and lived with a partner. PNC was reported by 93.7 % of women, with a significantly lower proportion among those undergoing post abortion hospitalization (41.8 %). Receipt of the PNC card was reported by 98.1 % of women who had PNC assistance, 74.9 % had the first PNC consultation until the 12th week of pregnancy and 74.5 % had the appropriate number of consultations. A higher proportion of women under the age of 20, of brown and black race/skin color, and with up to 11 years of schooling was observed in women with public financing, while in those with private financing, a higher proportion of women aged 35 or over, white, with 16 or more years of education and who lived with a partner was observed. A higher proportion of women with PNC, both postpartum and post abortion, with an early start and an adequate number of consultations was reported by women with private financing, as well as a lower proportion of PNC card receipt (Table 2).

**Table 2:** Sociodemographic characteristics and use of prenatal care. Rio de Janeiro/Brazil, 2021‒2023.

Characteristics	Total (*n* = 1923) (%)	Public financing (*n* = 1446) (%)	Private financing (*n* = 477) (%)
**Type of hospitalization**			
Childbirth care	91.6 (88.9‒93.7)	90.2 (87.8‒92.2)	95.8 (86.3‒98.8)
Abortion care	8.4 (6.3‒11.1)	9.8 (7.8‒12.2)	4.2 (1.2‒13.7)
**Maternal age (years)**			
< 20	9.7 (7.8‒12.2)	12.5 (10.7‒14.5)	1.4 (0.5‒3.8)
20 to 34	70.8 (68.5‒73.1)	73.0 (69.8‒75.9)	64.4(59.3‒69.1)
35 or more	19.4 (17.2‒21.8)	14.5 (12.9‒16.4)	34.2 (29.1‒39.7)
**Race/skin color**			
White	29.6 (25.0‒34.6)	22.9 (18.6‒27.8)	50.0 (43.8‒56.2)
Black	20.3 (16.7‒24.4)	23.9 (20.8‒27.4)	9.2 (6.5‒12.9)
Brown	50.1 (46.7‒53.5)	53.2 (48.7‒57.6)	40.8 (34.0‒48.0)
**Years of education**			
≤ 8	15.1 (12.5‒18.1)	19.8 (17.2‒22.6)	0.9 (0.5‒1.6)
9 to 11	28.0 (21.2‒35.9)	34.5 (28.5‒41.1)	8.1 (4.8‒13.4)
12 to 15	41.0 (35.5‒46.8)	40.5 (33.7‒47.7)	42.5 (34.6‒50.9)
16 or more	15.9 (11.3‒21.8)	5.2 (3.6‒7.2)	48.4 (37.3‒59.6)
**Live with a partner**	79.9 (75.6‒83.6)	75.4 (71.8‒78.7)	93.5 (89.6‒96.0)
**Pre-natal Care**	93.7 (91.8‒95.2)	92.1 (90.0‒93.8)	98.5 (95.9‒99.4)
Childbirth hospitalization	98.5 (97.5‒99.1)	98.0 (96.5‒98.8)	99.9 (98.9‒100)
Abortion hospitalization	41.8 (32.7‒51.4)	38.3 (30.2‒47.2)	66.3 (53.8‒76.9)
**Receipt of PNC card**^a^	98.1 (96.3‒99.0)	99.5 (98.1‒99.8)	94.2 (90.0‒96.6)
**Start of PNC until 12-weeks**^a^	74.9 (71.7‒77.8)	70.0 (66.4‒73.4)	93.9 (91.5‒95.6)
**Adequate n° of consultations**^a^	74.5 (71.6‒77.2)	69.2 (66.0‒72.3)	90.4 (84.9‒94.1)

PNC, Pre-Natal Care.

a Among women who received PNC (total = 1802, public = 1332, private = 470).

Among women with an available PNC card, 82.7 % had a record of a first syphilis test and 52.6 % had a record of a second test. The most used tests in the first testing were the combination of NTT+TT (43.6 %), followed by NTT (36.9 %) and TT (19.5 %). In the second testing, a higher proportion of NTT (58.9 %) and TT (24.4 %) was observed, with a lower proportion of NTT+TT (16.7 %). Women with public financing had a higher proportion of first testing (84.6 %) as well as a greater use of TT, alone or in combination (71 %). No significant differences for the second testing were observed (Table 3).

**Table 3:** Diagnosis of Gestational Syphilis. Rio de Janeiro/ Brazil, 2021‒2023.

Indicators	Total^a^ (*n* = 1353) (%)	Public financing (*n* = 1098) (%)	Private financing (*n* = 255) (%)
**First test for syphilis during PNC**	82.7 (77.3‒87.0)	84.6 (78.9‒89.0)	74.4 (67.7‒80.1)
**Type of test**^b^			
Treponemal (TT)	19.5 (11.2‒31.7)	21.7 (12.6‒34.8)	8.7 (4.6‒15.9)
Non-treponemal (NTT)	36.9 (21.6‒55.3)	29.0 (15.4‒47.8)	75.7 (62.1‒85.6)
Both (NTT+TT)	43.6 (33.9‒53.9)	49.3 (39.7‒59.0)	15.6 (8.1‒28.1)
**Second test for syphilis during PNC**^c^	52.6 (45.1‒59.9)	51.5 (42.3‒60.6)	57.2 (48.1‒65.8)
**Type of test**^d^			
Treponemal (TT)	24.4 (11.6‒44.3)	29.3 (14.8‒49.6)	6.3 (3.0‒12.8)
Non-treponemal (NTT)	58.9 (33.8‒80.0)	51.8 (26.9‒75.8)	85.5 (73.9‒92.5)
Both (NTT+TT)	16.7 (9.8‒27.0)	19.0 (11.4‒29.8)	8.2 (3.5‒17.9)
**Test for syphilis during hospitalization**^e^	81.6 (70.8‒89.0)	97.0 (93.1‒98.7)	35.1 (22.0‒50.8)
Childbirth care	81.3 (70.3‒88.9)	97.1 (92.9‒98.9)	36.2 (22.5‒52.5)
Abortion care	84.7 (64.7‒94.3)	95.3 (87.0‒98.4)	9.1 (3.1‒23.5)
**Type of test**^f^			
Treponemal (TT)	32.4 (18.1‒51.0)	36.1 (19.6‒56.7)	1.9 (0.5‒6.3)
Non-treponemal (NTT)	20.8 (9.2‒40.4)	13.2 (4.2‒ 34.9)	84.0 (67.5‒93.0)
Both (NTT+TT)	46.8 (26.0‒68.8)	50.7 (28.5‒72.7)	14.1 (5.3‒32.6)
**Diagnosis of Gestational Syphilis**^e^	14.5 (9.2‒22.2)	18.2 (12.3‒25.9)	3.6 (2.0‒6.4)
Confirmed^g^	54.4 (35.7‒71.9)	57.4(39.7‒73.5)	6.7 (0.9‒35.2)
Probable^g^	6.6 (2.2‒17.9)	4.2 (1.2‒13.7)	43.8 (15.0‒77.5)
Depends on clinical history^g^	25.0 (15.2‒38.2)	26.6 (15.3‒42.0)	0
Probable false-positive^g^	5.4 (1.8‒14.8)	4.1 (1.2‒13.1)	25.9 (9.2‒54.6)
Clinical diagnosis only^g^	8.6 (5.7‒12.9)	7.7 (4.5‒12.8)	23.6 (9.9‒46.6)
**Incident cases of Gestational Syphilis**^e^	3.0 (1.3‒6.6)	3.7 (1.7‒7.9)	0.7 (0.4‒1.5)
**Timing of syphilis diagnosis**^g^			
Pre-natal care	70.3 (61.4‒77.8)	68.8 (58.8‒77.3)	92.8 (56.5‒99.2)
Hospitalization (childbirth/abortion care)	29.7 (22.2‒38.6)	31.2 (22.7‒41.2)	7.2 (0.8‒43.5)

PNC, Pre-Natal Care.

a Among women with PNC card available.

b Among women with first test for syphilis (total = 1.119, public = 930, private = 189).

c Among women with gestational age at childbirth > 34-weeks (total = 1291, public = 1040, private = 251).

d Among women with gestational age at childbirth > 34-weeks and second test for syphilis (total = 679, public = 535, private = 144).

e Among all women (total = 1923, public = 1446, private = 477).

f Among women with test for syphilis during hospitalization (total = 1.570, public = 1.402, private = 167).

g Among women diagnosed with gestational syphilis (total = 280, public = 263, private = 17).

During hospitalization, testing for syphilis was recorded in 81.6 % of hospital records, with no differences regarding the type of hospitalization (childbirth or abortion care). A significant lower testing coverage was observed in women with private hospital financing, especially in hospitalizations for abortion care. Similar to testing during PNC, there was a predominant use of NTT tests in women with private financing, while in women with public financing, TT were more frequently used, alone or in combination with NTT tests (Table 3).

We identified 280 cases of GS syphilis during pregnancy (14.5 %; 95 % CI 9.2 %‒22.2 %), 263 of which were in women with public financing (18.2; 95 % CI 12.3 %‒25.9 %) and 17 in those with private financing (3.6 %; 95 % CI 2.0 %‒6.4 %) (Table 3). It is estimated that 57 (20.4 %) of these cases are incident cases. Most of the diagnoses were based on laboratory tests, with 54.4 % of cases classified as confirmed, 6.6 % as probable, 5.4 % as probable false-positives and 25 % dependent on clinical history. Only 8.6 % of cases were based solely on clinical information. Approximately one-third-of the cases were diagnosed during hospitalization (Table 3) and 2.1 % were coinfected with HIV.

Out of the total number of women diagnosed with GS, 28.3 % were not treated; 13.4 % were treated appropriately; 3.5 % inappropriately; 15.7 % were treated, but without information to assess adequacy; 9.4 % had no information about treatment; and 29.7 % were not treated because the diagnosis occurred during hospitalization. Among those diagnosed during PNC and who had a PNC card, the record of treatment (adequate, not adequate or without information to assess adequacy) was recorded in 53.6 % of the cases. As for the partner, only 3.8 % had a record of treatment; 57.1 % were not treated during PNC; 29.7 % were not treated because the diagnosis occurred during hospitalization and in 9.4 % of the cases, there was no information available about the partner's treatment.

When only women diagnosed with syphilis during PNC and who had a PNC card were analyzed (*n* = 170), it appears that the performance of the treatment was related to the laboratory result, with a higher proportion of pregnant women treated during PNC when the diagnosis of syphilis was “confirmed” or “probable”, in relation to “probable false-positive” cases, those “dependent on clinical history” and those with only a clinical diagnosis (Table 4).

**Table 4:** Treatment of Gestational Syphilis according to the type of diagnosis^a^.

Diagnosis of syphilis / Treatment	Not treated (%)	Treated (%)
Confirmed (*n* = 98)	39.2 (28.2‒51.5)	60.8 (48.5‒71.8)
Probable (*n* = 10)	31.7 (7.5‒72.8)	68.3 (27.2‒92.5)
Dependent on the clinical history (*n* = 36)	62.8 (26.5‒88.8)	37.2 (11.2‒73.5)
Probable false-positive (*n* = 5)	70.7 (23.3‒95.0)	29.3 (5.0‒76.7)
Clinical diagnosis (*n* = 21)	53.2 (34.2‒71.4)	46.8 (28.6‒65.8)
Total (*n* = 170)	46.4 (33.2‒60.2)	53.6 (39.8‒66.8)

a Among women with diagnosis of syphilis during PNC and with PNC card available.

The incidence of CS, considering only cases recorded in hospital records (*n* = 94), was 53.1 per 1000 LB (68.4 per 1000 LB in women with public financing, 9.7 per 1000 LB in those with private financing). The estimated incidence of CS was 78.2 per 1000 LB, with higher values in women with public financing. The vertical transmission rate was 33.5 % for registered cases and 50.1 % for estimated cases with no significant differences according to the source of hospital financing. For the two CS criteria used, most of the outcomes were live births at hospital discharge (100 % in women with private financing). In the estimated criteria, two cases of early fetal loss due to syphilis were identified. No cases of neonatal death due to CS were identified (Table 5).

**Table 5:** Treatment and outcomes of Gestational Syphilis. Rio de Janeiro/Brazil, 2021‒2023.

Indicators	Total (*n* = 1923) (%)	Public financing (*n* = 1446) (%)	Private financing (*n* = 477) (%)
**Pregnant woman treatment**^a^			
Not treated	28.3 (18.7‒40.4)	28.1 (18.0‒41.0)	31.1(10.8‒62.8)
Adequate treatment	13.4 (11.2‒16.0)	13.3 (11.4‒15.5)	15.0 (2.4‒55.9)
Inadequate treatment	3.5 (1.8‒6.9)	3.8 (1.8‒7.6)	0
No information to assess adequacy	15.7 (10.1‒23.5)	16.5 (10.4‒25.0)	3.4 (0.4‒24.2)
No information about treatment	9.4 (6.3‒13.8)	7.2 (5.1‒9.9)	43.3 (24.0‒65.0)
Diagnosis during hospitalization	29.7 (22.2‒38.6)	31.2 (22.7‒41.2)	7.2 (0.8‒43.5)
**Partner Treatment**^a^			
Yes	3.8 (0.9‒14.3)	3.9 (0.9‒15.6)	1.3 (0.1‒14.0)
No	57.1 (43.7‒69.6)	57.7 (44.0‒70.3)	48.2 (35.3‒61.3)
Diagnosis of GS during hospitalization	29.7 (22.2‒38.6)	31.2 (22.7‒41.2)	7.2 (0.8‒43.5)
No information	9.4 (6.3‒13.8)	7.2 (5.1‒9.9)	43.3 (24.0‒65.0)
**Incidence of Congenital Syphilis**^b^			
Registered cases (*n* = 94)	53.1 (27.3‒100.8)	68.4 (37.7‒120.9)	9.7 (4.2‒22.5)
Estimated cases (*n* = 140)	78.2 (39.5‒148.9)	100.5 (54.4‒178.5)	15.0 (7.7‒29.0)
**Vertical transmission rate**^a^			
Registered cases (*n* = 94)	33.5 (26.5‒41.4)	34.0 (27.0‒41.8)	26.2 (11.9‒48.3)
Estimated cases (*n* = 140)	50.1 (39.6‒60.5)	50.7 (40.1‒61.2)	40.5 (21.6‒62.8)
**Congenital Syphilis outcome (registered cases = 94)**			
Alive at hospital discharge	99.2 (92.7‒99.9)	99.2 (92.2‒99.9)	100 (100‒100)
Early fetal loss	0	0	0
Intermediate or late fetal loss	0.8 (0.1‒7.3)	0.8 (0.1‒7.8)	0
Neonatal death	0	0	0
**Congenital Syphilis outcome (estimated cases = 140)**			
Alive at hospital discharge	97.9 (91.0‒99.6)	97.8 (90.2‒99.6)	100 (100‒100)
Early fetal loss	1.2 (0.2‒7.9)	1.3 (0.2‒8.8)	0
Intermediate or late fetal loss	0.9 (0.2‒3.6)	0.9 (0.2‒3.9)	0
Neonatal death	0	0	0

GS, Gestational Syphilis.

a Among women with diagnosis of gestational syphilis (total = 280, public = 263, private = 17).

b Incidence of congenital syphilis per 1000 live biths. Estimated cases: registered cases + outcome of a pregnancy with a diagnosis of syphilis and no treatment or inadequate treatment.

## Discussion

Of the three process indicators indicated by the WHO for the management of GS and control of CS, only PNC reached the target of coverage ≥95 % among women hospitalized for childbirth care. Testing with a syphilis exam was <90 % in women with public and private financing and the treatment of infected pregnant women diagnosed during PNC was only registered in 53.6 % of the PNC cards, half of them without information about the type of treatment. The incidence of CS, considering only registered cases, was 53.1 per 1000 LB, a value 100 times higher than the WHO elimination target. Inequalities were observed according to the type of hospital financing, such as worse social condition, less access to PNC, as well as a higher rate of detection of GS and incidence of CS in women with public financing.

Compared to previous national studies and in the city of Rio de Janeiro, there was a worsening in coverage of the first testing for syphilis and an improvement in the second. In the “Birth in Brazil” study, carried out in 2011/2012, the coverage of the first and second testing in the Southeast region was 91.8 % and 44.5 %, respectively,^9^ while in the city of Rio de Janeiro, in a study carried out in 2007/2008, these values were 89.8 % and 35.3 %.^18^ In this study, first and second testing coverage was estimated at 82.7 % and 52.6 %. Similar to what was recorded in the National Information System of Notifiable Diseases (*Sistema de Informação de Agravos de Notificação* – SINAN), most of the tests were carried out using NTT and TT tests, although the frequency of NTT tests was higher in this study, especially in privately funded hospitalizations.

Data on treatment of pregnant women were not available in the “Birth in Brazil” study. In the city of Rio de Janeiro, in 2007/2008, 62 % of pregnant women diagnosed with syphilis reported having been treated, but the treatment record was only available on 21.4 % of the PNC cards.^19^ Data from SINAN^5^ demonstrates the prescription of at least one dose of benzathine penicillin for >90 % of pregnant women with GS reported in the RJS, a value much higher than that found in this study, which suggests problems in the quality of the registration of PNC cards.

The results of this study show a large increase in the prevalence of GS. In 2007/2008, the estimated GS prevalence in the city of Rio de Janeiro was of 1.9 % (95 % CI 1.3 %; 2.6 %),^6^ while in the Southeast region of Brazil, in 2011/2012, this value was 1.03 % (95 % CI 0.75 %; 1.41 %).^9^ In this study, the estimated prevalence, considering any reactive test result or registered clinical diagnosis, the same criteria adopted in previous studies, was 14.5 %, almost ten times higher.

The increase in the prevalence of GS cases is consistent with the notification data recorded in SINAN, which also reveals an increase in the detection rate in the country from 2012 onwards.^5^ However, the GS detection rate estimated in this study (139. 4 per 1000 LB, data not shown in table) was more than double the detection rate recorded in the RJS in SINAN in 2022, of 69.7 per 1000,^5^ which suggests failures in case notification. The GS detection rate in SINAN is close to the prevalence of cases classified as “confirmed” and “probable” in this study, which may suggest that only these cases are being treated and reported as GS cases. The lower proportion of treatment carried out in women classified as “probable false-positive” and in those dependent on the assessment of clinical history reinforces this hypothesis. The notification coverage of GS cases in the RJS in the period 2007‒2018 was estimated at 83.13 % (95 % CI 74.77 %‒93.74 %),^20^ that is, with an estimated underreporting of approximately 17 %. The main hypotheses for underreporting of GS were barriers to access PNC services and testing for syphilis.^20^ Our results suggest a higher rate of underreporting and the possibility that even cases among tested women may be underreported if healthcare professionals do not consider the test results as indicative of syphilis infection.

The incidence of CS of 53.1 per 1000 LB represents an increase of 14 times in relation to the “Birth in Brazil” study (3.81/1000 LB in the Southeast region in 2011/2012)^10^ and 9 times in relation to a study in the city of Rio de Janeiro in 2007/2008 (6 per 1000),^6^ with a similar VT rate, close to 35 %.^6^^,^^10^ However, the value observed in this study is >2 times higher than the incidence of CS reported to SINAN (23.0 per 1000 LB in 2022),^5^ also suggesting flaws in CS notification. The underreporting may be even higher if we consider the estimated cases of CS, which included cases considered probable due to inadequate or non-performed treatment, in addition to those recorded in medical records. The estimated cases would increase the incidence of CS by three times, when compared to cases reported in SINAN, with VT rate of 50 %, and the identification of 2 cases of abortions due to syphilis. Failures in recording abortions due to syphilis had already been identified in previous studies,^6^^,^^8^ as well as underreporting of CS cases.^7^^,^^8^ Although problems in the recording of PNC cards may be overestimating these cases, the results of this study suggest probable flaws in the identification and notification of cases in childbirth and abortion care services that should be investigated in specific studies.

Our results reveal the missed opportunities for reducing CS in the country, such as the failure to carry out testing during PNC, especially the second test, resulting in one-third-of the cases being diagnosed only during hospitalization for childbirth care; the failure to carry out testing during hospitalization for childbirth/abortion in almost 20 % of hospitalizations, with worse performance in privately funded hospitalizations; and failures in the treatment of women and partners. Even though the partner's treatment is no longer considered a criterion for defining a case of CS, its implementation is important to reduce the transmission of syphilis in the general population.^21^

This study has some limitations. Women admitted to hospitals with <100 births/year and those with home or public births were not included and, therefore, the results do not apply to these women. The adopted classification of hospitalization according to the type of financing classifies some women admitted to private hospitals affiliated with the Unified Health System (SUS) as public hospitalizations, making it impossible to assess possible differences between women treated in these private hospitals or in public hospitals. The absence of a record of exams and treatment on the PNC card was considered as a lack of performance. Although this may have overestimated the proportion of untested and untreated women, we understand that the PNC card is the main link between outpatient and hospital care and should be adequately completed. The lack of information on diagnosis and previous treatment also made it difficult to evaluate women whose classification of infection and need for treatment depended on this information, which corresponds to 25 % of cases. As data from exams performed in the three moments were available, we presented a proposal for an algorithm for classifying GS cases. However, this assessment is subject to the quality of the exams recorded on the PNC card and hospital records. Likewise, the diagnosis of CS cases depended on recording in the hospital records, with no evaluation of the cases recorded by the research team.

## Conclusion

Congenital syphilis is an important public health problem in Brazil and in the Rio de Janeiro state. In this study, several opportunities for improving the management of GS were identified, both in publically and privately funded hospitalizations, aiming to reduce VT and the incidence of CS. Women with public financing for childbirth and abortion care presented greater social vulnerability and a higher prevalence of GS and incidence of CS, and should be a priority target of control strategies. These should include reducing syphilis in the general population through the promotion of safe sex and diagnosis and treatment of syphilis infection, as well as the appropriate management of gestational syphilis through timely diagnosis and treatment of women and sexual partners. Initiatives to improve notification of both diseases are necessary.

## Funding

This work was supported by the Secretaria de Ciência, Tecnologia e Inovação e do Complexo Econômico-Industrial da Saúde – SECTICS/MS [grant n° 25000.174797/2023-14], Conselho Nacional de Desenvolvimento Científico e Tecnológico (CNPq) [grant n° 443766/2018-5], Fundação Oswaldo Cruz Programa Inova Geração de Conhecimento [grant number VPPCB-007-FIO-18), The Newton Fund Institutional Links On Impact And Evidence-Based Policies (Health And Neglected Diseases) framework [grant n° 25380.002237/2020-81] and Fundação de Amparo à Pesquisa do Estado do Rio de Janeiro (FAPERJ) /7ª Edição do programa Pesquisa para o SUS: gestão compartilhada em saúde – PPSUS [grant n° E-26/210.463/2021].

## Conflicts of interest

The authors declare no conflicts of interest.
